# The Sepsis ImmunoScore Predicts Sepsis, Mortality, and Deterioration Better than Clinical Scores and Widely Available Biomarkers

**DOI:** 10.3390/diagnostics16131962

**Published:** 2026-06-24

**Authors:** Gregory L. Watson, Lincoln C. Updike, Carlos G. López-Espina, Akhil Bhargava, Lee A. Schmalz, Shah Khan, Dennys S. Urdiales, Matthew D. Sims, Ashok V. Palagiri, Adrian D. Haimovich, Alon Dagan, Benjamin P. Davis, Karen C. White, Paul A. Gurbel, Stockton M. Mayer, Anwaruddin Syed, Sihai Dave Zhao, Ruoqing Zhu, Rashid Bashir, Nathan I. Shapiro, Bobby Reddy

**Affiliations:** 1 Prenosis, Inc., Chicago, IL 60616, USA; lincoln.updike@prenosis.com (L.C.U.); carlos.lopez-espina@prenosis.com (C.G.L.-E.); akhil.bhargava@prenosis.com (A.B.); lee.schmalz@prenosis.com (L.A.S.); shah.khan@prenosis.com (S.K.); dennys.urdiales@prenosis.com (D.S.U.); bobby.reddy.jr@prenosis.com (B.R.J.); 2 Department of Medicine, Section of Infectious Diseases and International Medicine, Corewell Health William Beaumont University Hospital, Royal Oak, MI 48073, USA; matthew.sims@corewellhealth.org; 3 Departments of Internal Medicine and Foundational Medical Studies, Oakland University William Beaumont School of Medicine, Rochester, MI 48309, USA; 4 Mercy Hospital, St. Louis, MO 63141, USA; ashok.palagiri@mercy.net; 5 Department of Emergency Medicine, Beth Israel Deaconess Medical Center, Boston, MA 02215, USA; nshapiro@bidmc.harvard.edu (N.I.S.); ahaimovi@bidmc.harvard.edu (A.D.H.); adagan@bidmc.harvard.edu (A.D.); 6 Carle Foundation Hospital, Urbana, IL 61801, USA; benjamin.davis@carle.com (B.P.D.); karen.white@carle.com (K.C.W.); 7 Sinai Hospital of Baltimore, LifeBridge Health, Baltimore, MD 21215, USA; pgurbel@lifebridgehealth.org; 8 Jesse Brown VA Medical Center, Chicago, IL 60612, USA; stockton.mayer@va.gov; 9 OSF Saint Francis Medical Center, Peoria, IL 61637, USA; anwaruddin.k.syed@osfhealthcare.org; 10 Department of Statistics, University of Illinois at Urbana-Champaign, Urbana, IL 61801, USA; dave.zhao@gmail.com (S.D.Z.); rqzhu@illinois.edu (R.Z.); 11 Department of Bioengineering, University of Illinois at Urbana-Champaign, Urbana, IL 61801, USA; rbashir@illinois.edu; 12 Department of Emergency Medicine, Harvard Medical School, Boston, MA 02115, USA

**Keywords:** sepsis, diagnostic techniques, artificial intelligence, predictive algorithms, precision medicine

## Abstract

**Background:** Early and accurate risk stratification of patients suspected of serious infection is essential for improving outcomes, but existing diagnostic and predictive tools have limited accuracy. The objective was to compare the performance of an FDA-authorized AI diagnostic test, the Sepsis ImmunoScore, against widely available biomarkers and clinical tools for diagnosis of sepsis and prediction of in-hospital mortality and intensive care unit (ICU) admission. **Methods:** This multicenter observational study included 6027 adult patients suspected of infection across 7 U.S. hospital sites. The Sepsis ImmunoScore’s predictive performance was compared to the sequential organ failure assessment (SOFA) score, procalcitonin (PCT), C-reactive protein (CRP), Systemic Inflammatory Response Syndrome (SIRS) score, National Early Warning Score (NEWS), and quick SOFA (qSOFA). Primary outcomes included sepsis as defined by Sepsis-3 criteria, in-hospital mortality, and ICU admission. Predictive accuracy was assessed using area under the receiver operating characteristic curve (AUC), and 95% confidence intervals were generated and hypothesis testing conducted using the bootstrap method. **Results:** The Sepsis ImmunoScore demonstrated statistically significant superior performance across all outcomes. For sepsis prediction, the Sepsis ImmunoScore achieved an AUC of 0.82, compared to SOFA (0.72), procalcitonin (PCT) (0.70), C-reactive protein (CRP) (0.61), SIRS (0.59), NEWS (0.69), and qSOFA (0.67). For in-hospital mortality prediction, the Sepsis ImmunoScore achieved an AUC of 0.80, outperforming SOFA (0.72), PCT (0.67), CRP (0.58), SIRS (0.60), NEWS (0.72), and qSOFA (0.69). For ICU admission, the Sepsis ImmunoScore reached an AUC of 0.74, superior to SOFA (0.63), PCT (0.64), CRP (0.54), SIRS (0.60), NEWS (0.70), and qSOFA (0.65). All differences between the Sepsis ImmunoScore and comparators were statistically significant. **Conclusions:** The Sepsis ImmunoScore significantly improved predictive accuracy for sepsis, in-hospital mortality, and ICU admission compared to six conventional clinical scores and biomarkers. This AI-based tool may enhance risk stratification and clinical decision-making, potentially leading to more timely sepsis interventions and improved outcomes.

## 1. Introduction

Sepsis is a serious, common, and heterogeneous medical syndrome caused by a dysregulated host response to infection [1]. It is a major cause of morbidity and mortality in the United States and worldwide, globally accounting for an estimated 48.9 million cases and 11.0 million deaths in 2017 [2]. In the United States it causes roughly 1.7 million adult hospitalizations and 270,000 deaths each year [3]. Early source control can improve patient outcomes, but prompt recognition of sepsis is challenging, because presentations are heterogeneous and overlap with non-infectious illness, and because the choice and timing of antimicrobial and supportive therapy must often be made before confirmatory data are available [4]. Widely used clinical scores and single biomarkers have limited and variable accuracy in this setting, which represents a public health gap. There remains an unmet clinical need for risk assessment tools that enable clinicians to promptly identify patients at risk of sepsis and consequent deleterious outcomes.

Many proposed approaches exist, including laboratory tests, sepsis biomarkers, clinical scores, and artificial intelligence (AI) tools, but none has gained universal acceptance in clinical practice. A recent addition is the Sepsis ImmunoScore^®^, which became the first AI diagnostic for sepsis authorized by the Food and Drug Administration (FDA) in 2024 [5,6]. The Sepsis ImmunoScore is similar to traditional laboratory tests in that it is only assessed for a patient when it is ordered by a clinician, and a result is returned regardless of whether it predicts the patient to be at low or high risk. This differs from most AI approaches to sepsis, which are passive algorithms running continuously in the background and produce pop-up alerts for patients only when particular high-risk criteria are met. In contrast, the Sepsis ImmunoScore is not a passive, population-wide screen; it is a clinician-ordered test that performs risk stratification within the population already identified by clinical suspicion of serious infection (i.e., a blood culture order). The present study evaluates its performance in precisely that intended-use population.

Despite a proliferation of AI and machine learning tools for sepsis, their real-world value remains uncertain, and reported performance is often more impressive than informative. A recent overview of 28 clinical AI sepsis studies found markedly mixed results, with recurring high false-positive rates and a frequent absence of external validation [7]. Several factors inflate headline metrics. First, discrimination is commonly reported in large, unselected inpatient or continuously monitored populations in which the great majority of patients are plainly not septic; in such low-prevalence settings a high area under the curve (AUC) can coexist with a positive predictive value too low to be clinically useful. A continuously running alert for flagging patients at risk of sepsis, for example, has reported a positive predictive value of less than 12% despite related publications reporting high discrimination [8,9], meaning most alerts are false. Second, high specificity is often achieved at the expense of sensitivity: a widely cited random-forest model attained 98% specificity but only 26% sensitivity—missing roughly three of every four cases—and, deployed prospectively, produced no significant improvement in mortality, length of stay, or ICU transfer [10]. Third, most models are validated only retrospectively within a single health system, and when subjected to true external validation they frequently fail; the widely deployed Epic Sepsis Model achieved an AUC of just 0.63 with low sensitivity and frequent false alarms on independent validation, yielding no added clinical benefit [11]. These experiences have prompted the conclusion that individual centers may need to retrain such models locally [7].

Against this backdrop, the Sepsis ImmunoScore is distinct: it is the first AI sepsis diagnostic to receive FDA De Novo authorization [5,6], and unlike the passive, continuously running alerts that dominate the literature, it is a clinician-ordered test applied to patients already under evaluation for serious infection. The present study was designed to provide a head-to-head comparison of the Sepsis ImmunoScore against the standard clinical scores and biomarkers it is meant to complement in an independent multicenter cohort.

The objective of this study was to compare the predictive performance of the Sepsis ImmunoScore against six salient comparators in a large prospective observational study of patients suspected of serious infection. We selected the sequential organ failure assessment (SOFA) score, procalcitonin (PCT), C-reactive protein (CRP), the Systemic Inflammatory Response Syndrome (SIRS) score, the National Early Warning Score (NEWS), and the quick SOFA (qSOFA) score for this comparison.

The SOFA score is a clinical score of organ dysfunction that has been used to predict mortality, especially for ICU patients, and is part of the Sepsis-3 definition [1,12,13]. PCT and CRP are FDA-approved biomarkers of infection that are widely available in clinical laboratories. PCT is a host-response biomarker upregulated by bacterial endotoxins and proinflammatory cytokines, including interleukin-6 and tumor necrosis factor-α. It has been found to be associated with mortality in patients with sepsis and patients with community-acquired pneumonia [14,15]. CRP is a non-specific marker of inflammation that is associated with mortality in patients with sepsis [16]. SIRS reflects an exaggerated response to infection, surgery, acute inflammation, or another stressor and is evaluated using cutoffs for body temperature, heart rate, respiratory rate, and leukocyte count [17]. A SIRS score of 2 or higher on the first day of hospitalization has been linked to prolonged ICU admission, a greater likelihood of developing multiple organ dysfunction syndrome (MODS), and increased need for vasopressors and mechanical ventilation [17]. NEWSs include measures such as supplemental oxygen usage, vital signs, and level of consciousness; the measure has demonstrated the ability to predict death, ICU admission, and cardiac arrest within 24 h and is broadly used for acute care assessments in the United Kingdom and elsewhere [18]. The qSOFA uses any two of its three criteria (Glasgow coma scale, respiratory rate and blood pressure) to identify patients at an increased risk of sepsis, prolonged ICU stay, or in-hospital mortality [1].

We evaluated the performance of the Sepsis ImmunoScore and these six comparators for predicting sepsis, in-hospital mortality, and admission to the ICU in patients suspected of serious infection. These tools are often used in triaging patients to determine whether they merit further testing, escalation in therapy, or admission to the ICU. Furthermore, they can be integrated into electronic medical record (EMR) systems to automatically trigger sepsis alerts, which frequently include SIRS and/or SOFA criteria [19,20,21,22]. Unlike PCT, CRP, and the Sepsis ImmunoScore, which all must be specifically ordered by a clinician, an EMR-based sepsis alert system can run constantly in the background, generating pop-up notices for all patients meeting the system’s criteria for possible sepsis. While these alerts aim to increase early sepsis identification and intervention, they often have very high false-positive rates that result in alert fatigue and even complete dismissal of the warnings by clinicians [20]. Some alert systems show modest success in improving hospital compliance with certain SEP-1 bundle criteria (a set of actions that must be taken within the first 3–6 h following sepsis identification in order to receive reimbursement from the Centers for Medicare and Medicaid Services), but most automated alerts do not improve mortality outcomes [20]. Other AI-based automated sepsis tools often incorporate criteria from these clinical scoring systems in addition to data such as lab values or even clinical notes. These tools were not evaluated as comparators in this study, because they function predominantly as passive background alerts rather than clinician-ordered diagnostics, and most are proprietary and not FDA-authorized.

## 2. Methods

### 2.1. Study Design

This was a retrospective analysis of prospectively collected blood samples and linked EMR data from a multicenter, observational study of hospitalized patients with suspected infection. Subjects were enrolled at 1 of 7 participating U.S. hospitals between March 2018 and June 2025. The study was approved by the ethics boards of each institution under a waiver of informed consent, except for OSF Saint Francis Medical Center, which required informed consent.

### 2.2. Study Population

The study enrolled hospitalized adult patients (aged 18 years and older) receiving care in any hospital department with a suspected serious infection, as indicated by a clinician’s decision to order a blood culture. To qualify, participants needed to have a lithium-heparin (Li-Hep) plasma sample drawn and collected and to have a valid Sepsis ImmunoScore result when retrospectively assessed at the time of their first blood culture order. There were no other exclusion criteria. The Sepsis ImmunoScore algorithm was developed using a separate patient cohort [5]; the study population did not include patients on whom the model was trained.

### 2.3. Data Collection

Remnant blood samples were prospectively collected from all eligible patients as part of an ongoing biobank run by Prenosis, Inc. (Chicago, IL, USA). The biobank performed PCT and CRP testing on the Li-Hep remnants using the Luminex Assay Platform. Prior to transport, a research coordinator removed direct identifiers and assigned each sample a unique specimen code (pseudonymization). Clinical data were extracted from the EMR via direct offline extraction, stripped of direct identifiers, and subsequently linked to biobanked samples using the specimen code. The coded linkage key was held separately, and the dataset used for the analysis contained no protected health information. Extracted data included demographic variables, ICD-10 diagnostic codes, medication records, vital signs, and results from routine laboratory tests such as blood chemistry and lactate levels. Comorbidities were classified according to the Charlson Comorbidity Index, ICD-10-Clinical Modification (CM) codes, and definitions from the National Cancer Institute’s Comorbidity Index and the Surveillance, Epidemiology, and End Results (SEER) program [23]. Immunocompromised status was determined using ICD-10-CM criteria established by the Agency for Healthcare Research and Quality [24].

Further details on data anonymization, retrieval, classification, and analysis are described in separate publications [5,25]. A Sepsis ImmunoScore was generated for each patient as an eligibility criterion; SOFA, SIRS, NEWS, and qSOFA scores were generated for each patient retrospectively using EMR data at the time of analysis.

### 2.4. The Sepsis ImmunoScore

The Sepsis ImmunoScore is a locked, FDA-authorized (De Novo DEN230036) calibrated random-forest model that integrates up to 22 objective patient parameters (demographics, vital signs, hematology and chemistry values, and the sepsis biomarkers PCT and CRP) to output a sepsis risk probability and one of four fixed risk categories. It functions like a diagnostic test that can be ordered by the treating clinician within a patient’s electronic health record (EHR). Twelve inputs are required for a valid result: systolic and diastolic blood pressure, temperature, respiratory rate, heart rate, oxygen saturation, white blood cell and platelet counts, blood urea nitrogen, creatinine, procalcitonin, and C-reactive protein; and ten are optional when available: age, lymphocyte and neutrophil counts, potassium, chloride, total CO_2_, sodium, albumin, bilirubin, and lactate. The model was deliberately restricted to objective measurements of patient biology, excluding subjective assessments and interventions to limit overfitting and improve generalizability. Algorithm development, time period for valid measures, selection decisions when multiple measurements are available, training and validation cohorts, threshold derivation, and feature importance (interventional SHAP values) are described in detail in the development study [5]. The clinical interpretability of these SHAP values for the Sepsis ImmunoScore has been separately validated [26]. The model was applied here exactly as authorized, without modification.

**Figure 1:**
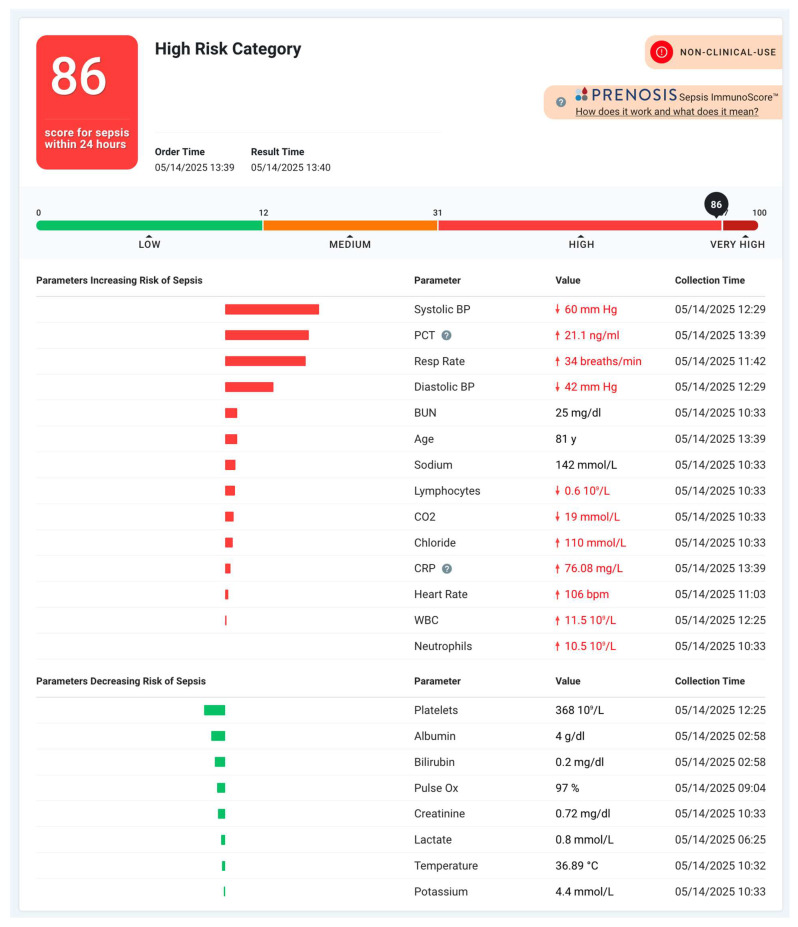
Sample Sepsis ImmunoScore Result Report. Parameter values are depicted in red if they fall outside clinically normal ranges with an arrow pointing up or down to indicate whether they are elevated or decreased relative to normal.

The pivotal study found that the Sepsis ImmunoScore was also predictive of other negative outcomes, including in-hospital mortality, length of hospital stay, intensive care unit (ICU) admission, mechanical ventilation, and vasopressor medication use in this same patient population [5]. The approved algorithm is locked to avoid overfitting the model, meaning it is not adjusted for individual institutions, and provides a user-friendly results display explaining a patient’s risk for each outcome and the clinical or laboratory data that weighed most heavily in calculating their score (Figure 1).

### 2.5. Study Outcomes

There were three outcomes of interest: sepsis diagnosis, in-hospital mortality, and ICU admission (in patients not in the ICU when blood cultures were first ordered). Sepsis within 24 h of the time of blood culture was assessed using an automated Sepsis-3 label determined retrospectively from each patient’s EMR [3]. In-hospital mortality and ICU admission status were determined from standardized data (Fast Healthcare Interoperability Resources) in the patient EMR.

### 2.6. Statistical Analysis

Predictive performance was assessed using the area under the receiver operating characteristic curve (AUC). We used 1000 bootstrapped samples to estimate 95% confidence intervals and conduct hypothesis tests for the difference in AUC between the Sepsis ImmunoScore and each of the comparators [27]. A total of 18 hypothesis tests were conducted for the primary analysis. We used the Bonferroni correction to account for multiple testing, rejecting each null hypothesis if the *p*-value was less than 0.05/18 [28].

To assess clinical utility, we performed decision-curve analysis for each outcome, computing net benefit across a range of clinically relevant threshold probabilities (0–75% for sepsis, 0–25% for in-hospital mortality, and 0–50% for ICU admission). We compared the Sepsis ImmunoScore against each comparator and with the treat-all and treat-none strategies. Because net benefit is evaluated on a predicted-probability scale, each comparator score and biomarker was transformed to a predicted risk of the corresponding outcome using univariable logistic regression. The Sepsis ImmunoScore was used on its native probability scale for sepsis prediction and was mapped to in-hospital mortality and ICU admission by univariate logistic regression. Analyses were conducted in R using the dcurves package.

To address the structural dependence of the Sepsis-3 label on the SOFA score, we performed a sensitivity analysis repeating the sepsis-prediction comparison using a SOFA-independent reference standard: severe sepsis identified from ICD-10-CM diagnostic codes (R65.20, R65.21, T81.12). AUCs and bootstrap 95% confidence intervals for the Sepsis ImmunoScore and all comparators were estimated as in the primary analysis.

To evaluate fairness, we assessed Sepsis ImmunoScore discrimination within subgroups defined by sex, age, and race/ethnicity for each outcome, with bootstrap 95% confidence intervals, and tested for between-subgroup differences in AUC. To be conservative in identifying differences in performance between subgroups, we did not account for multiple testing when comparing subgroups.

To characterize performance across the four fixed Sepsis ImmunoScore risk categories (low, medium, high, very high), we reported the observed predictive value and likelihood ratio within each category for each outcome. We also reported additional diagnostic metric (sensitivity, specificity, PPV, NPV, and F1) at each of the three category boundaries, interpreting each boundary as a candidate binary decision threshold. Calibration of the Sepsis ImmunoScore was assessed graphically and quantified using the Brier score and Expected Calibration Error.

To assess robustness of the locked model without altering it, we evaluated Sepsis ImmunoScore discrimination stratified by enrollment era (2018–2021 vs. 2022–2025) and by clinical site. Because the algorithm is FDA-authorized and locked, it was not re-fit for these analyses; the same model was applied across all strata.

The analysis was conducted using R statistical software version 4.4.1 (R Foundation for Statistical Computing; Vienna, Austria).

## 3. Results

There were a total of 6027 patient encounters with valid Sepsis ImmunoScore results during the study period (Table 1). Mean age was 64.2 years (SD 17.5 years) with a slight male predominance (52.9%), and two-thirds (65.5%) of the population were White. High-risk comorbidities were common, and more than one-third of the sample (35.2%) met the automated Sepsis-3-based criteria for sepsis within 24 h of the first ordered blood culture. Mean length of stay was nearly 10 days, and 28.0% of patients were transferred to the ICU, with 6.6% dying during their hospital stay.

**Figure 2:**
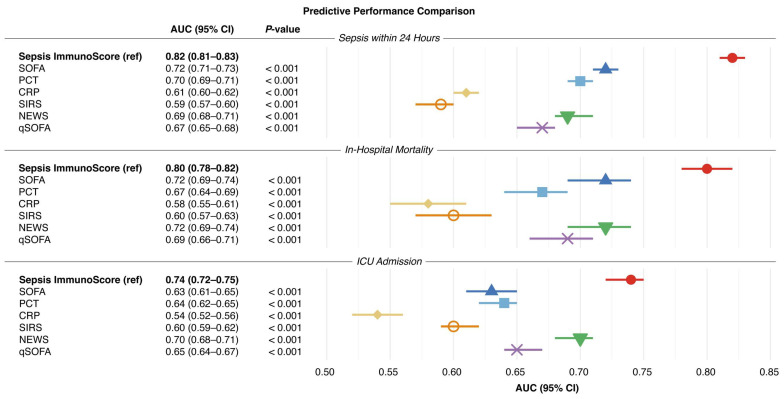
Comparative AUCs of Predictive Tools Across Study Outcomes.

The AUC of the Sepsis ImmunoScore (0.82; 95% CI: 0.81–0.83) for predicting sepsis within 24 h of first blood culture order was significantly greater than the AUCs of all six comparators (*p* < 0.001) (Figure 2). Likewise, the AUC of the Sepsis ImmunoScore for in-hospital mortality (0.80; 95% CI: 0.78–0.82) and admission to the ICU (0.74, 95% CI: 0.72–0.75) were both significantly better than those of all comparators (*p* < 0.001) (Figure 2).

Sepsis ImmunoScore performance was stable over time and across sites. Discrimination was unchanged between enrollment periods for sepsis (AUC 0.82 in both 2018–2021 and 2022–2025) and was maintained or marginally higher in the later period for in-hospital mortality and ICU admission (Supplementary Table S1). Across the seven participating hospitals, the Sepsis ImmunoScore had the highest mean site-level AUC for sepsis (0.80; range 0.71–0.84) and the highest minimum site-level AUC of any tool evaluated (0.71), exceeding the mean site-level AUC of every comparator and remaining the top performer even at the least favorable site (Supplementary Table S2).

**Figure 3:**
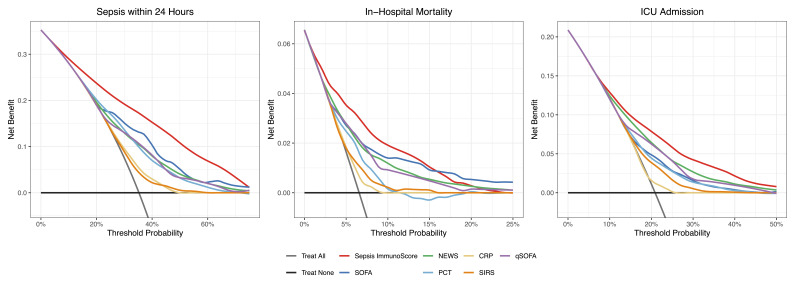
Decision-curve analysis of the Sepsis ImmunoScore and comparators for (**left**) sepsis within 24 h, (**center**) in-hospital mortality, and (**right**) ICU admission. Curves show net benefit (net true positives per patient) versus threshold probability. “Treat all” and “treat none” are reference strategies. Comparator scores and biomarkers were converted to predicted risks by univariable logistic regression.

Decision-curve analysis (Figure 3) showed that the Sepsis ImmunoScore provided the highest net benefit of all evaluated strategies across the clinically relevant threshold ranges for sepsis, in-hospital mortality, and ICU admission. Its advantage over the comparators widened as the threshold probability increased, and it maintained net benefit above both the treat-all and treat-none strategies throughout the displayed ranges. By contrast, the treat-all strategy became net-harmful beyond each outcome’s prevalence, and CRP, PCT and SIRS fell to the net benefit of treating no one over part of the mortality threshold range. At a threshold probability of 20% for sepsis, use of the Sepsis ImmunoScore rather than PCT (the strongest comparator at this threshold) corresponded to 3.6 additional true cases identified per 100 patients with no increase in unnecessary interventions.

Discrimination of the Sepsis ImmunoScore was consistent across subgroups for sepsis prediction (AUCs 0.80–0.83, Supplementary Table S3). For the secondary outcomes, discrimination was modestly higher in women than men for in-hospital mortality (0.83 vs. 0.77) and higher in younger than older patients for both in-hospital mortality (0.84 vs. 0.76) and ICU admission (0.76 vs. 0.72), with non-overlapping confidence intervals; estimates did not differ by race or ethnicity for any outcome. The Sepsis ImmunoScore maintained good discrimination across all subgroups (all AUCs ≥ 0.72).

In a sensitivity analysis using severe sepsis defined by ICD-10 diagnostic codes as the outcome, the Sepsis ImmunoScore again demonstrated the highest discrimination (AUC 0.80; 95% CI 0.78–0.81), significantly exceeding all comparators, including SOFA (0.64; 95% CI 0.63–0.66) (Supplementary Table S4). The Sepsis ImmunoScore’s advantage over SOFA was larger under this SOFA-independent label than under the Sepsis-3 label.

Observed risk increased monotonically across the four Sepsis ImmunoScore categories for all outcomes (Supplementary Table S5). For sepsis within 24 h, the observed rate rose from 8% in the low-risk category (likelihood ratio 0.15) to 84% in the very-high category (likelihood ratio 9.76); corresponding gradients were 1% to 24% for in-hospital mortality and 8% to 58% for ICU admission. At the medium-to-high boundary, the Sepsis ImmunoScore identified sepsis with a sensitivity of 0.77 and specificity of 0.71, and at the highest boundary specificity reached 0.99 (Supplementary Table S6).

The Sepsis ImmunoScore was well calibrated for sepsis prediction, the tool’s native output. Predicted and observed risk agreed closely across the range of predicted probabilities (Supplementary Figure S1), with an Expected Calibration Error of 0.038 (mean absolute deviation between predicted and observed risk of approximately 3.8 percentage points) and a Brier score of 0.165, below the no-information value of approximately 0.23 implied by the sepsis prevalence. The monotonic concordance between risk categories and observed event rates across all three outcomes (Supplementary Table S5) provides complementary evidence that the locked model stratifies risk appropriately in this independent cohort.

## 4. Discussion

In this large, multicenter study we found that the Sepsis ImmunoScore significantly outperformed six widely available biomarkers and clinical scoring tools for the prediction of sepsis diagnosis, in-hospital mortality, and clinical deterioration (as indicated by admission to the ICU) for patients suspected of serious infection. This potentially has several clinically meaningful ramifications.

Delayed recognition of septic shock may delay administration of antimicrobials and interventions that support tissue perfusion (e.g., fluid resuscitation and vasopressors), with a resulting increase in the risk of negative outcomes [29,30,31,32]. The connection between rapid administration of broad-spectrum antibiotics and reduced mortality in patients suspected of sepsis has been repeatedly described [29,33,34,35]. More accurate predictions may improve risk stratification and prompt more timely interventions, which in turn promise to improve outcomes.

For patients with signs of severe illness, a test result suggesting a low likelihood of sepsis may direct clinician investigation toward alternative explanations for patient symptoms, aiding in differential diagnosis. Improved risk stratification may also make the Sepsis ImmunoScore useful for other clinical decisions, such as patient disposition. Confirming that a patient is at a low risk of sepsis or clinical deterioration may, when physician judgment aligns, support the decision to de-escalate therapy, refrain from use of antimicrobials, keep a patient on a standard inpatient floor, or even discharge a patient home. An enhanced risk prediction tool that can prompt further investigations for sick patients, keep higher-level beds open for more at-risk patients, and send healthy patients home sooner could result in more efficient use of healthcare resources, reduced wait times for ICU admission for the sickest patients, better antimicrobial stewardship, and improved patient safety. Beyond superior discrimination, decision-curve analysis indicated that acting on the Sepsis ImmunoScore would yield greater net clinical benefit than acting on standard scores, biomarkers, or default strategies across the threshold ranges clinicians are likely to use.

Early and more accurate identification of sepsis may have financial impacts as well. Improved diagnosis can aid hospitals in SEP-1 bundle compliance by enabling prompt initiation of the required interventions and assessments. Results from an FDA-approved diagnostic can facilitate accurate patient coding and provide documentation for sepsis-related billing and claims. Together, improved SEP-1 compliance and appropriate coding have substantial potential to impact hospital reimbursement for the complex management of sepsis patients.

The Sepsis ImmunoScore differs from other FDA-authorized diagnostic tools for sepsis in its superior performance and holistic assessment of patient risk for sepsis, ICU admission, and in-hospital mortality. Unlike many FDA-approved diagnostic tools for infection that focus on limited markers, such as PCT, CRP, monocyte distribution width [36], leukocyte biophysical properties [37], or immune-related gene expression [38], the Sepsis ImmunoScore integrates multidimensional inputs, including PCT, CRP, vital signs, and clinical data commonly used in scoring systems like SOFA, SIRS, and NEWS. This holistic integration of patient-specific factors enables the Sepsis ImmunoScore to reflect a comprehensive view of patient risk, enhancing the predictive power beyond that of individual markers or scores.

Unlike passive surveillance tools that continuously monitor EMR data and trigger alerts, many of which employ the clinical scores or biomarkers studied here [20,39], the Sepsis ImmunoScore functions as a clinician-ordered diagnostic test; its score places patients into one of four discrete risk categories and is intended for use in conjunction with clinical assessments [40]. Rather than supplant background alerts, we propose that the Sepsis ImmunoScore could complement existing alert systems by reducing false positives and alert fatigue. For example, hospitals could automatically order the Sepsis ImmunoScore along with requisite labs for patients flagged by background alerts, using its patient-specific results report to support decision-making regarding disposition and escalation of care.

The Sepsis ImmunoScore also performed consistently across the seven participating hospitals, and the site-level results highlight a reliability advantage that pooled estimates may obscure. At their least favorable site, several comparators discriminated little better than chance: SIRS, qSOFA, CRP, and SOFA all fell to AUCs of approximately 0.52–0.58, whereas the Sepsis ImmunoScore’s lowest site-level AUC was 0.71. Because any individual hospital experiences the performance of the tool at its own site rather than the multi-site average, this consistency is clinically consequential: a score that approaches chance at some institutions offers little dependable decision support there, regardless of however well it performs on average. The comparatively narrow site-to-site range of the Sepsis ImmunoScore (0.71–0.84) suggests its discrimination is more robust to differences across hospitals.

Our findings also extend the evidence available for this tool. Prior performance data derived from its development and validation study [5]. That study did not benchmark the Sepsis ImmunoScore head-to-head against the standard scores and biomarkers used at the bedside, which the present study does in a larger multicenter cohort. Reported performance across AI sepsis models is strikingly heterogeneous, a recent review of 28 studies described AUCs ranging from approximately 0.61 to above 0.99 [7], but these values are not directly comparable to ours, reflecting different prediction tasks (forecasting onset hours in advance versus point-of-care diagnostic risk assessment), populations (ICU, emergency department, or all monitored inpatients), and sepsis definitions. High discrimination in large, unselected, continuously monitored populations, in which most patients are plainly not septic, can coexist with clinically inadequate positive predictive value, and several widely deployed systems have failed external validation [11]. Evaluated here in its intended-use population against the comparators clinicians actually rely on, the Sepsis ImmunoScore showed consistent, statistically significant superiority across all three outcomes (AUC 0.82 for sepsis within 24 h, 0.80 for in-hospital mortality, and 0.74 for ICU admission).

Discrimination for sepsis, the authorized indication, was uniform across sex, race, ethnicity, and age. For the secondary outcomes of mortality and ICU admission, discrimination was somewhat lower in older patients and, for mortality, in men. These differences were modest and the tool retained good discrimination in all subgroups, but they merit consideration: the lower discrimination for mortality in older patients may reflect the higher baseline burden of competing comorbidities and non-sepsis mortality in this group, which complicates prediction. The analysis assessed discrimination rather than the full range of algorithmic fairness criteria, including subgroup calibration, which warrants dedicated study.

One potential concern with the primary analysis is the partial dependence of the Sepsis-3 reference standard on SOFA. Because the Sepsis ImmunoScore shares several physiological and laboratory inputs with SOFA, this raises the possibility that its apparent superiority reflects this overlap rather than genuine added value. Several lines of evidence argue against this. First, the Sepsis ImmunoScore also outperformed all comparators for in-hospital mortality and ICU admission, objective outcomes independent of any sepsis definition. Second, we deliberately included SOFA as a comparator because, as a constituent of the Sepsis-3 label, it is the most favorable and hardest-to-beat comparator for this sepsis outcome. Third, in a sensitivity analysis using a SOFA-independent severe-sepsis (ICD-10) label, the Sepsis ImmunoScore retained high discrimination (AUC 0.80; significantly better than all six comparators) while SOFA’s fell substantially to 0.64. These findings suggest that the Sepsis ImmunoScore’s superiority is robust to the limitations of the Sepsis-3 definition, while the performance of SOFA appears to be partially an artifact of the Sepsis-3 label construction. This further solidifies the evidence that the Sepsis ImmunoScore outperforms the relevant comparators.

### Limitations

There are several limitations to this study. First, it is possible that the sample of patients included from the 7 participating hospitals do not reflect patients elsewhere, and we cannot guarantee generalizability of our findings. We attempted to minimize this possibility with bootstrap analysis and the conservative Bonferroni correction to limit the likelihood of spurious findings; the consistent trend of superior performance of the Sepsis ImmunoScore across all outcomes was expected due to the multifaceted, inclusive design of the algorithm. While the automated determination of sepsis assessed in this study has been widely used [3], it may result in some classification error compared to gold standard chart review. Likewise, missing data from EMRs could have caused misclassification or missed comorbidities.

While we selected widely available, well-known tools and biomarkers for sepsis prediction, we could not compare the Sepsis ImmunoScore to proprietary AI-based algorithms that may have more competitive AUCs for the outcomes of interest. We also did not compare the Sepsis ImmunoScore to passive sepsis alert systems that evaluate every patient regardless of infection-related indicators. By design, the Sepsis ImmunoScore must be specifically ordered by a clinician like any other diagnostic test. This is intended to ensure that sufficient information is obtained to make a reliable assessment of the patient’s risk (via prompts to order any additional tests that the score requires). As such, it cannot identify septic patients for whom the test is not ordered. Instead, it serves two primary purposes: to provide documented support for a practitioner’s judgment about a patient’s status (cases very likely to be or not to be sepsis), or to aid in differential diagnosis and clinical decision-making when the clinical picture is unclear. A well-designed prospective study will be required to demonstrate the clinical impact of the Sepsis ImmunoScore in practice and to determine in which patient subpopulations its report is most informative and influential.

The Sepsis ImmunoScore requires laboratory inputs, including PCT and CRP, and its result is therefore available only once these inputs return. In settings without rapid assays, this introduces a turnaround time of potentially several hours that could attenuate the benefit of earlier intervention. This limitation is intrinsic to any biomarker-informed tool, including the PCT and CRP comparators studied here. To mitigate this limitation within clinical workflows, we recommend that any required labs, including PCT and CRP, be ordered simultaneously with the Sepsis ImmunoScore. With this approach the score does not add delay beyond that of the laboratory results themselves. Nonetheless, the time-to-result and its real-world impact on intervention timing should be quantified in a prospective implementation study.

Because the Sepsis ImmunoScore is a locked, FDA-authorized device, we did not perform leave-one-hospital-out or train/test temporal re-derivations, which would evaluate models other than the deployed device; instead, we assessed robustness of the fixed model across sites and enrollment eras, observing stable performance in both.

## 5. Conclusions

While sepsis is still a nebulous construct with heterogenous presentations and a variable disease course, the ability of the Sepsis ImmunoScore to predict mortality and objective metrics of clinical deterioration provides evidence that it reflects important underlying biological risks. As ongoing clinical research refines our understanding of sepsis into one of an overarching problem—extreme, dysregulated immune response—with distinct pathophysiological subtypes [41,42,43,44], we believe holistic, biology-based tools will continue to outperform symptom-based tools, isolated biomarkers, and institutional practice-based indicators in sepsis diagnosis. Just as we have seen cancer outcomes improve as the scientific understanding of cancer evolved, refined definitions of sepsis according to subtypes could provide the opportunity to develop and deploy personalized, effective therapies targeting the key biological underpinnings of each patient’s disease.

While we make progress toward that reality, this study provides evidence that the Sepsis ImmunoScore outperforms numerous existing sepsis evaluation tools. It can be easily integrated into the EMR and offers an FDA-authorized diagnostic to determine a patient’s imminent risk of sepsis as well as a prediction of in-hospital mortality, ICU admission, length of stay, and likelihood of requiring mechanical ventilation or vasopressors. Regardless of whether hospitals implement the Sepsis ImmunoScore as a stand-alone diagnostic or paired with background warning systems, our analysis suggests this AI-based algorithm offers excellent diagnostic accuracy for sepsis. By extension, the Sepsis ImmunoScore has the potential to impact clinical practice and hospital resources through more rapid and appropriate triage, informed treatment decisions, opportunities for greater SEP-1 bundle compliance, and improved documentation for coding and claims.

## Figures and Tables

**Table 1:** Baseline Data and Clinical Outcomes.

Patient Characteristics (*N* = 6027)	Patient Encounters *n* (%)
Clinical site	
William Beaumont University Hospital—Royal Oak, MI, USA	1993 (33.1%)
Mercy Health—St. Louis, MO, USA	1733 (28.8%)
Beth Israel Deaconess Medical Center—Boston, MA, USA	1105 (18.3%)
Sinai Hospital—Baltimore, MD, USA	712 (11.8%)
OSF Saint Francis Medical Center—Peoria, IL, USA	207 (3.4%)
Carle Foundation Hospital—Urbana, IL, USA	149 (2.5%)
Jesse Brown VA Medical Center—Chicago, IL, USA	128 (2.1%)
Age (mean [±SD])	64.2 (17.5)
Male	3185 (52.9%)
Female	2842 (47.1%)
Race	
American Indian or Alaska Native	17 (0.3%)
Asian	129 (2.1%)
Black or African American	1553 (25.8%)
Native Hawaiian or Other Pacific Islander	6 (0.1%)
Unknown	372 (6.2%)
White	3950 (65.5%)
Ethnicity	
Hispanic or Latino Ethnicity	464 (7.7%)
Not Hispanic or Latino Ethnicity	5563 (92.3%)
High-risk comorbidities ^a^	
Acute myocardial infarction	308 (5.1%)
History of myocardial infarction	439 (7.3%)
Congestive heart failure	1688 (28.0%)
Cerebrovascular disease	626 (10.4%)
Chronic obstructive pulmonary disease	1473 (24.4%)
Dementia	666 (11.1%)
Paralysis	243 (4.0%)
Diabetes	1457 (24.2%)
Diabetes with complications	1308 (21.7%)
Renal disease	1862 (30.9%)
Mild liver disease	627 (10.4%)
Moderate and severe liver disease	253 (4.2%)
Peptic ulcer disease	130 (2.2%)
Rheumatologic disease	284 (4.7%)
AIDS	42 (0.7%)
Immunocompromised	1489 (24.7%)
COVID-19	502 (8.3%)
Clinical outcomes of interest	
Sepsis-3 within 24 h	2124 (35.2%)
In-hospital mortality	395 (6.6%)
ICU transfer	1145 (20.9%) ^b^
Placement of mechanical ventilation	726 (12.0%)
Administration of vasopressor	952 (15.8%)
Mean length of stay, in days (±SD)	9.5 (13.1)

^a^ Comorbidities were classified according to the Charlson Comorbidity Index, ICD-10-Clinical Modification (CM) codes, and definitions from the National Cancer Institute’s Comorbidity Index and the Surveillance, Epidemiology, and End Results (SEER) program. ^b^ Calculated only for patients not in the ICU at the time of first blood culture order (*n* = 5485).

## Data Availability

The data that support the findings of this study are available from Prenosis, Inc., but restrictions apply to the availability of these data, which were used under license for the current study, and so are not publicly available. Data are however available from the authors upon reasonable request and with permission of Prenosis, Inc.

## Supplementary Materials

The following supporting information can be downloaded at: https://www.mdpi.com/article/10.3390/diagnostics16131962/s1, Table S1. Sepsis ImmunoScore Performance by Time Period. Table S2. Per-Site Sepsis Prediction Performance. Table S3. Discrimination of the Sepsis ImmunoScore by Patient Subgroup. Table S4. Predictive Tool Performance on Severe-Sepsis ICD Label. Table S5. Sepsis ImmunoScore Risk Category Performance Indicators. Table S6. Diagnostic Metrics at Risk Category Boundary Cutoffs. Figure S1. Calibration of the Sepsis ImmunoScore.

## Abbreviations

AI	Artificial Intelligence
AUC	Area Under the Receiver Operating Characteristic Curve
CRP	C-reactive Protein
EHR	Electronic Health Record
EMR	Electronic Medical Record
FDA	Food and Drug Administration
ICD-10-CM	International Classification of Diseases, Tenth Revision, Clinical Modification
ICU	Intensive Care Unit
ML	Machine Learning
MODS	Multiple Organ Dysfunction Syndrome
NEWS	National Early Warning Score
PCT	Procalcitonin
qSOFA	Quick Sequential Organ Failure Assessment
SD	Standard Deviation
SEER	Surveillance, Epidemiology, and End Results
SEP-1	Severe Sepsis and Septic Shock Management
SIRS	Systemic Inflammatory Response Syndrome
SOFA	Sequential Organ Failure Assessment
