# Supplementary material for: The Sepsis ImmunoScore Predicts Sepsis, Mortality, and Deterioration Better than Clinical Scores and Widely Available Biomarkers
Source: Diagnostics (Basel). 2026 Jun 24;16(13):1962. doi: 10.3390/diagnostics16131962 (PMC13360300; doi:10.3390/diagnostics16131962)
Supplement: Supplementary file 1 [file diagnostics-16-01962-s001.zip › diagnostics-4301543-supplementary.pdf]

Table S1. Sepsis ImmunoScore Performance by Time Period.

Outcome	2018–2021	2022–2025
Sepsis within 24 h	0.82 (0.80–0.83)	0.82 (0.80–0.83)
In-Hospital Mortality	0.78 (0.76–0.81)	0.81 (0.78–0.84)
ICU Admission	0.73 (0.70–0.75)	0.76 (0.73–0.78)

Table S2. Per-Site Sepsis Prediction Performance.

Predictive Tool	Mean Site AUC	Minimum Site AUC	Maximum Site AUC
Sepsis ImmunoScore	0.80	0.71	0.84
PCT	0.71	0.63	0.78
NEWS	0.67	0.58	0.74
qSOFA	0.63	0.52	0.73
CRP	0.60	0.54	0.71
SOFA	0.70	0.54	0.79
SIRS	0.58	0.54	0.63

Table S3. Discrimination of the Sepsis ImmunoScore by Patient Subgroup.

Subgroup	Sepsis Within 24 h	In-Hospital Mortality	ICU Admission
Gender			
Female	0.82 (0.81–0.84)	0.83 (0.80–0.85)	0.75 (0.72–0.77)
Male	0.81 (0.79–0.82)	0.77 (0.74–0.79)	0.72 (0.70–0.75)
Race			
White	0.81 (0.80–0.83)	0.79 (0.76–0.81)	0.75 (0.72–0.78)
Black	0.82 (0.80–0.85)	0.82 (0.79–0.86)	0.73 (0.71–0.75)
Ethnicity			
Hispanic or Latino	0.83 (0.76–0.90)	0.85 (0.72–0.95)	0.80 (0.72–0.87)
Not Hispanic or Latino	0.82 (0.81–0.83)	0.79 (0.77–0.81)	0.74 (0.72–0.75)
Age			
18–64	0.83 (0.82–0.85)	0.84 (0.80–0.86)	0.76 (0.74–0.78)
65+	0.80 (0.78–0.81)	0.76 (0.73–0.78)	0.72 (0.70–0.74)

Table S4. Predictive Tool Performance on Severe-Sepsis ICD Label.

Predictive Tool	AUC (95% CI) for Severe-Sepsis ICD Label
Sepsis ImmunoScore	0.80 (0.78–0.81)
PCT	0.75 (0.74–0.77)
NEWS	0.71 (0.69–0.72)
qSOFA	0.67 (0.65–0.68)
CRP	0.65 (0.63–0.67)
SOFA	0.64 (0.62–0.66)
SIRS	0.62 (0.61–0.64)

Table S5. Sepsis ImmunoScore Risk Category Performance Indicators.

Outcome	Risk Category	Total Patients	Patients with Event	PV	LR
Sepsis within 24 h	Low	1885	146	0.08	0.15
	Medium	1360	335	0.25	0.60
	High	2435	1351	0.55	2.29
	Very High	347	292	0.84	9.76
In-Hospital Mortality	Low	1885	13	0.01	0.10

	Medium	1360	43	0.03	0.47
	High	2435	256	0.11	1.68
	Very High	347	83	0.24	4.48
ICU Admission	Low	1827	139	0.08	0.31
	Medium	1276	193	0.15	0.68
	High	2126	664	0.31	1.72
	Very High	256	149	0.58	5.28

Table S6. Diagnostic Metrics at Risk Category Boundary Cutoffs.

Outcome	Cutoff	Sensitivity	Specificity	PPV	NPV	F1
Sepsis within 24 h	12.2	0.93	0.45	0.48	0.92	0.63
	30.6	0.77	0.71	0.59	0.85	0.67
	87.2	0.14	0.99	0.84	0.68	0.24
In-Hospital Mortality	12.2	0.97	0.33	0.09	0.99	0.17
	30.6	0.86	0.57	0.12	0.98	0.21
	87.2	0.21	0.95	0.24	0.95	0.22
ICU Admission	12.2	0.88	0.39	0.28	0.92	0.42
	30.6	0.71	0.64	0.34	0.89	0.46
	87.2	0.13	0.98	0.58	0.81	0.21

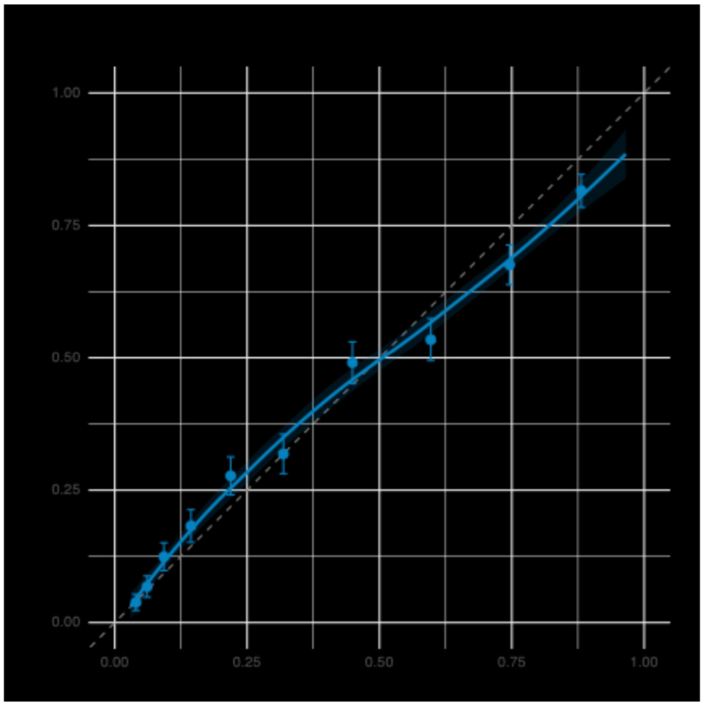

Figure S1. Calibration of the Sepsis ImmunoScore.
